# Is the Psychopathic Brain an Artifact of Coding Bias? A Systematic Review

**DOI:** 10.3389/fpsyg.2021.654336

**Published:** 2021-04-12

**Authors:** Jarkko Jalava, Stephanie Griffiths, Rasmus Rosenberg Larsen, B. Emma Alcott

**Affiliations:** ^1^Department of Interdisciplinary Studies, Okanagan College, Penticton, BC, Canada; ^2^Department of Psychology, Okanagan College, Penticton, BC, Canada; ^3^Werklund School of Education, University of Calgary, Calgary, AB, Canada; ^4^Forensic Science Program and Department of Philosophy, University of Toronto Mississauga, Mississauga, ON, Canada; ^5^Irving K. Barber School of Arts and Sciences, University of British Columbia, Kelowna, BC, Canada

**Keywords:** psychopath, PCL-R, sMRI, review studies, systematic review, coding bias

## Abstract

Questionable research practices are a well-recognized problem in psychology. Coding bias, or the tendency of review studies to disproportionately cite positive findings from original research, has received comparatively little attention. Coding bias is more likely to occur when original research, such as neuroimaging, includes large numbers of effects, and is most concerning in applied contexts. We evaluated coding bias in reviews of structural magnetic resonance imaging (sMRI) studies of PCL-R psychopathy. We used PRISMA guidelines to locate all relevant original sMRI studies and reviews. The proportion of null-findings cited in reviews was significantly lower than those reported in original research, indicating coding bias. Coding bias was not affected by publication date or review design. Reviews recommending forensic applications—such as treatment amenability or reduced criminal responsibility—were no more accurate than purely theoretical reviews. Coding bias may have contributed to a perception that structural brain abnormalities in psychopaths are more consistent than they actually are, and by extension that sMRI findings are suitable for forensic application. We discuss possible sources for the pervasive coding bias we observed, and we provide recommendations to counteract this bias in review studies. Until coding bias is addressed, we argue that this literature should not inform conclusions about psychopaths' neurobiology, especially in forensic contexts.

## Introduction

Psychopathy, as assessed by the *Hare Psychopathy Checklist-Revised* (PCL-R), is a psychiatric construct associated with affective and interpersonal abnormalities as well as antisocial behavior (Hare, 2003). In the criminal justice system, PCL-R evaluations have been used to inform decisions about such things as sentencing, institutional placement, parole, juvenile transfers, and treatment amenability (Gacono, 2016; Patrick, 2018). Neuroimaging studies have found structural and functional abnormalities in psychopaths, and as a result many researchers view psychopathy as a neurobiological disorder (e.g., Blair, 2013; Lushing et al., 2016; Sethi et al., 2018; Yang and Raine, 2018). Some authors have argued that these abnormalities might be taken into account when determining psychopaths' criminal responsibility (e.g., Blair, 2008; Anderson and Kiehl, 2013; Raine, 2019), amenability to neurosurgery or pharmacological treatment (e.g., De Ridder et al., 2009; Glenn and Raine, 2009), and when trying to predict their future dangerousness (e.g., Nadelhoffer et al., 2012; Umbach et al., 2015). Neuroimaging evidence on psychopaths has already been presented in court, including in death penalty hearings [e.g., State v. Brian, 2009; State v. Jerome, 2015; see also Denno (2015)].

However, the reliability of psychological data—and by extension their readiness for application—has come under increasing scrutiny. For decades, psychological research has been criticized for producing an unrealistically high proportion of positive findings (Sterling, 1959; Greenwald, 1975; Sterling et al., 1995; Fanelli, 2012). Recent studies describe a particularly acute problem in cognitive neuroscience where, depending on the year, up to 90% of all published findings have been positive (Fanelli, 2012). The high prevalence of positive findings is concerning for two reasons. First, as neither neuroimaging methods nor psychological tests have particularly high reliability, a significant proportion of reported findings may be false positives. Second, there is a well-recognized set of biases favoring positive findings, which may be eliminating true null-findings from the literature (Vul et al., 2009; Wager et al., 2009; Button et al., 2013; Nugent et al., 2013; Szucs and Ioannidis, 2017; Vul and Pashler, 2017). The biases toward positive findings include the file drawer problem (only studies with positive findings are submitted to journals; Rosenthal, 1979), publication and reporting bias (journals are more likely to publish and authors to report positive than null-findings; Jennings and Van Horn, 2012; David et al., 2013, 2018; Dwan et al., 2013; Ioannidis et al., 2014), and p-hacking (researchers use flexible data analyses to produce positive finding; Nelson et al., 2018).

These and other Questionable Research Practices [QRPs; see John et al. (2012)] in original research may also skew review studies and meta-analyses. A recent study comparing effects from meta-analyses and large-scale replication studies in psychology—the latter avoiding QRPs through pre-registration—found that meta-analytic effect sizes were indeed significantly larger (Kvarven et al., 2020). Some researchers have argued that reviews and meta-analyses may actually amplify biases in original research. This could be for at least two reasons. First, since biases in original studies tend to be systematic—toward fewer nulls—aggregating the studies in meta-analyses will only intensify the biases (Nelson et al., 2018). Second, reviews and meta-analyses (henceforth, “review literature”) may have QRPs of their own. These include funding bias (e.g., Jørgensen et al., 2006; Bes-Rastrollo et al., 2013; Mandrioli et al., 2016), citation bias [Duyx et al., 2017; but not always; see Nuijten et al. (2020)], spin (e.g., Drucker et al., 2016; Yavchitz et al., 2016; McGrath et al., 2017), and *post-hoc* changes to registered review protocols (Silagy et al., 2002).

An additional QRP in review literature that has received far less attention is the so-called *coding bias* (also known as data *extraction bias*; Petticrew and Roberts, 2008). Coding bias refers to the decisions reviewers make about which data to extract from a study. Coding bias in review literature is analogous to reporting bias in original research—just as an original study can highlight positive findings in abstracts while burying nulls in supplemental tables or not reporting them at all, a reviewer can do the same by selectively coding positive findings (coding bias is different from citation bias, as the latter only addresses biases in study choice, not in within-study effects). Coding bias is most likely to occur in fields such as cognitive neuroscience where a single study can report a large number of effects, and where reviewers therefore enjoy many of the same kinds of “degrees of freedom” as original researchers (Müller et al., 2018). Although coding bias has received some attention (Orwin and Cordray, 1985; Wortman and Bryant, 1985; Petticrew and Roberts, 2008), it has not been systematically evaluated. In this paper, we examine coding bias in neuroimaging research on psychopathy. We define coding bias as a selective extraction of positive findings from original studies by authors of review literature. To measure coding bias, we compared the proportion of null-findings in meta-analyses and review studies to the proportion of null-findings in original research. We adopted “Preferred Reporting Items for Systematic Reviews and Meta-Analyses” (PRISMA) guidelines for locating original studies and review literature (Liberati et al., 2009), and we used an expert consensus extraction strategy for all effects. We also examined whether the agreement between original and review literatures varied as a function of publication date or type of review.

We focused on two clearly defined parameters: psychopathy measured by the *Hare Psychopathy Checklist-Revised* (PCL-R) or its *Screening Version* (PCL:SV) (Hart et al., 1995; Hare, 2003) and brain abnormalities as described by structural magnetic resonance imaging (sMRI) data. The PCL-R is considered the standard measure for psychopathy in forensic settings (Hare, 2003, 2016; Glenn and Raine, 2008). We focused on sMRI as opposed to functional (fMRI) studies, as fMRI studies employ a wide range of tasks that make between-study comparisons difficult. Also, review studies often fail to include descriptions of task conditions in their summaries of fMRI findings, making it difficult to know exactly which task a reviewer is referring to.

## Methods

### Literature Search Inclusion and Exclusion Criteria

#### Original sMRI Studies

Studies were included if they reported either case-control or correlational sMRI data on PCL-R or PCL:SV defined psychopathy samples. Exclusion criteria were (i) studies published in language other than English, (ii) studies conducted on youth or adolescents, and (iii) studies that did not report sufficient detail on PCL-R or PCL:SV scores (e.g., not reporting total scores).

#### Review Literature

Meta-analyses and review studies were included if their stated or implied purpose was to review neuroimaging research on psychopathy, and included sMRI data on PCL-R or PCL:SV defined psychopathy. Exclusion criteria were (i) studies published in language other than English, (ii) studies reviewing data only on youth or adolescents, (iii) studies that did not report sufficient detail on PCL-R or PCL:SV scores (e.g., not reporting total scores), (iv) studies published by any of the current authors to avoid the possibility of bias.

### Search Strategy

#### Original sMRI Studies

**Figure 1:**
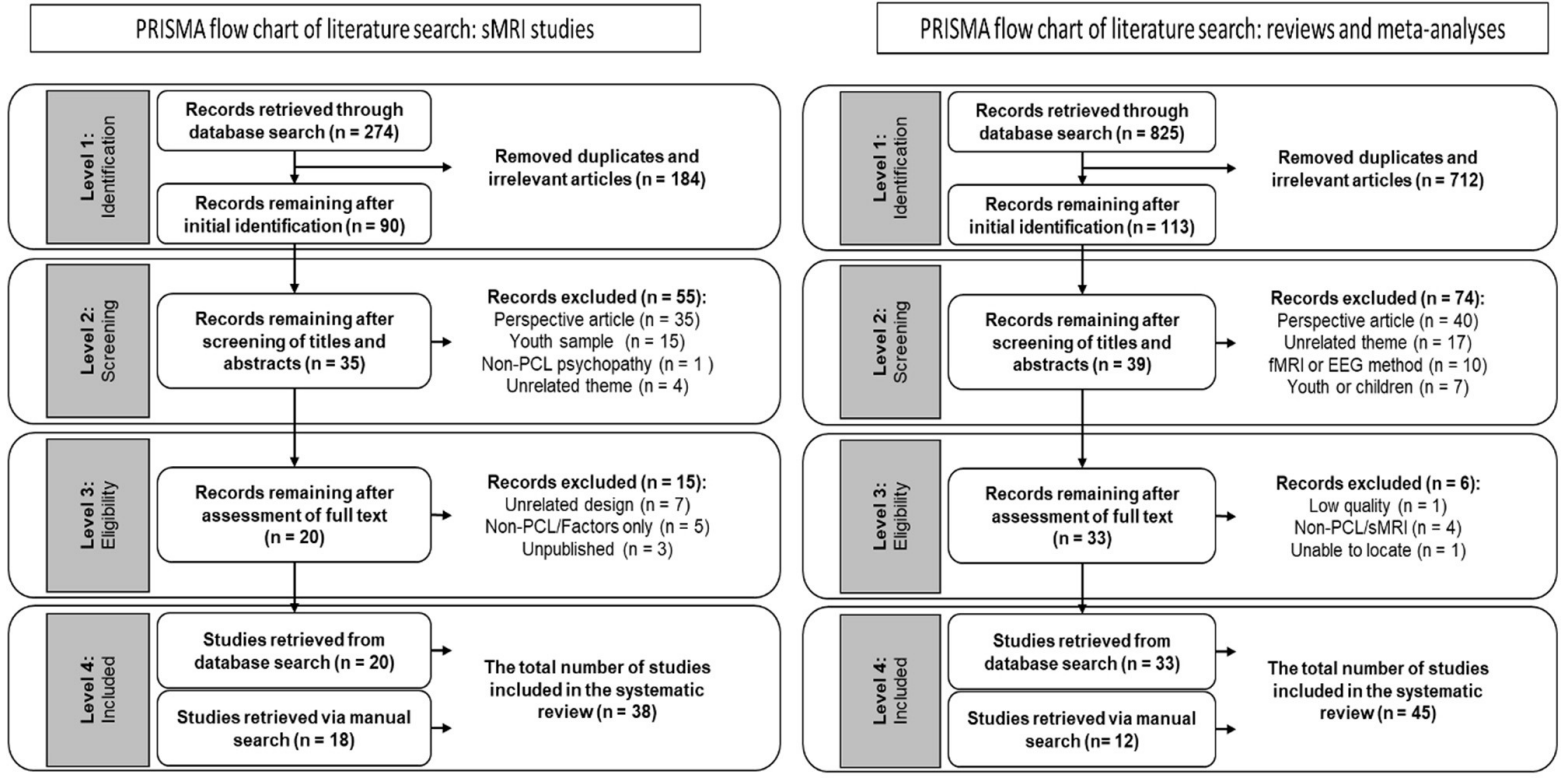
PRISMA flow charts.

We conducted a full-text, English-language only PRISMA search in the years 1995–2020, using the keyword sets (Psychopathic OR Psychopathy OR psychopath OR pcl^*^) AND (neuro^*^ OR brain) AND (smri OR structural). The initial search yielded 274 records (Medline n = 124; PsycINFO *n* = 150). We excluded 184 records that were duplicates and/or thematically irrelevant (i.e., the keywords or titles clearly suggested the article was unrelated to our search topic). The identified 90 records were exported to Endnote X9 (Clarivate Analytics), where we scanned the titles and abstracts to determine their relevance. At this step we excluded 55 articles. We then examined the full text for the remaining articles, and excluded 15 studies that either (a) used unrelated design, (b) used a measure other than the PCL-R, (c) did not report PCL-R total score, or (d) were unpublished.^1^ Twenty records were retained for analysis in our study (for workflow, see Figure 1). Finally, we manually scanned recent neuroimaging review studies on psychopathy to determine if our initial search missed any relevant publications. This manual scan identified an additional 18 records, resulting in a total of 38 studies retained for our analysis. We excluded unpublished studies, even when cited in review literature, as they were not available for coding.

#### Review Literature

We conducted a full-text, English-language only PRISMA search in the years 1995–2020, using the keyword sets (Psychopathic OR Psychopathy OR psychopath OR pcl^*^) AND (neuro^*^ OR brain) AND (meta^*^ OR review). The initial search yielded 825 records (Medline n = 534; PsycINFO *n* = 291). We excluded 712 records that were duplicates and/or thematically irrelevant (i.e., the keywords or titles clearly suggested the article was unrelated to our search topic). We retained 113 records, which we exported to Endnote X9 (Clarivate Analytics), where we scanned the titles and abstracts to determine their relevance. At this step we excluded 74 articles. We then examined the full text for the remaining 39 articles, and excluded six studies that either (a) did not disclose sufficient information on the studies reviewed, (b) did not use the PCL-R, (c) did not use sMRI, or (d) could not be located.^2^ Thirty three records were retained for analysis in our study (for workflow, see Figure 1). Finally, we conducted a manual scan (reference sections of review studies and recommendations from manuscript reviewers) to determine if our initial search missed any relevant publications. This manual scan identified an additional 12 relevant studies, resulting in a total of 45 studies retained for our analysis.

### Data Coding

#### Null-Findings in Original sMRI Studies

We followed a systematic coding strategy for null- and positive findings [see Griffiths and Jalava (2017)]. We first examined the percentage of null-findings in the original 38 research studies by recording all regions of interest (ROIs) identified in the introduction section of each article. If statistically significant regions beyond the ROIs were reported in results section, these regions were added to the total ROIs. We then recorded all ROIs in the results section by examining test statistics and/or *p*-values to identify statistically significant findings. Null-findings were identified either by test statistics and/or *p*-values or by missing results for ROIs that had been clearly identified in the introduction. In case of whole-brain analysis/exploratory research supplementary tables were used to identify null-rejections and null-findings. We followed reporting patterns of original studies with each reported effect counting as an ROI. In between-group designs, only group comparisons were reported (i.e., no correlations between foci and PCL-R score). In research designs using more than two groups, all group comparisons were recorded. Psychopathy groups included any subjects indicated as psychopathic (e.g., “medium psychopathy” and “high psychopathy” or “successful psychopath” and “unsuccessful psychopath”). We did not report on regions recorded as manipulation checks or methodological controls. White and gray matter and lateralized findings were included as separate data points. Finally, when relevant, only corrected findings were reported [e.g., controlling for multiple comparisons, small volumes, and drug use; see Müller et al. (2018)]. Two of the authors (J.J., S.G.) reviewed the number of foci. Any disagreements were resolved by a third author (R.R.L.).

#### Null-Findings in Review Literature

We adopted the following coding strategy for the 45 studies included: We examined the number of foci described either as a positive finding (increased or decreased volume, abnormal shape, etc.) or null-finding for PCL-R or PCL:SV total scores or for all factors. We included findings only when a clear comparison (e.g., psychopaths vs. control) or correlations with PCL-R or PCL:SV score in specified regions was reported. White and gray matter, and right and left findings were each scored separately. The same approach was used for different structural measures (e.g., volume, thickness, etc.). If a finding was described as “bilateral” or referred to in plural (e.g., amygdalae, gyri, nuclei, etc.), it was coded accordingly as two separate findings. If a finding in an individual study referred to “volumes” (e.g., amygdala volumes), it was coded as two separate findings. However, if “volumes” referred to more than one study, it was coded as one finding per study. Three of the authors (J.J., S.G., and E.A.) reviewed the number of foci. Any disagreements were resolved in a fourth review by two of the authors (J.J. and S.G.). For more details on the coding process and examples of it, see Appendix in Supplementary Material.

## Results

### Proportion of Null-Findings in Original sMRI Studies

The above method yielded the following ratios: Of the 791 effects recorded in 38 original sMRI studies 64.10% (507 out of 791) were null-findings, and 35.90% (284 out of 791) were positive findings (see Table 1). We examined the data for outliers, and identified one study (Boccardi et al., 2011) that reported a total of 312 comparisons, out of which 130 were positive findings. When we excluded these 312 comparisons, the proportion of null-findings across the remaining 37 studies was 67.85% (325 out of 479), indicating that the single study with a large number of comparisons did not unduly affect the proportion of null-findings.

**Table 1:** Summary of sMRI effects.

**Original sMRI studies**	**Study details**		**Number of effects**		
	**ROI identification**	**Statistical corrections**	**Total ROIs (N)**	**Reject % (N)**	**Null %(N)**
Raine et al. (2000)	Introduction and results text, tables	Multivariate statistical controls for drug use	4	50% (2)	50% (2)
Laakso et al. (2001)	Introduction and results text, tables	Hippocampal slices at 5% volume intervals; Bonferroni correction of p-values reported for PCL-R Total score-volume correlations	40	5% (2)	95% (38)
Laakso et al. (2002)	Introduction and results text, tables	Group design to control for drug use	10	0% (0)	100% (10)
Raine et al. (2003)	Introduction and results text, tables	MANCOVA for confounds	5	60% (3)	40% (2)
Raine et al. (2004)	Introduction and results text, tables	Bonferroni correction for multiple comparisons	6	33.33% (2)	66.67% (4)
Yang et al. (2005)	Introduction and results text, tables	Statistical control (ANOVA) for confounds	6	33.33% (2)	66.67% (4)
Schiltz et al. (2007)	Introduction and results text	Voxel-wise thresholding (*p* < 0.001 uncorrected)	1	0% (0)	100% (1)
de Oliveira-Souza et al. (2008)	Introduction and results text, tables	FDR (*p* < 0.05) for all ROIs	18	22.22% (4)	77.78% (14)
Müller et al. (2008)	Introduction and results text, tables	Corrected for multiple comparisons across all ROIs	12	8.33% (1)	91.67% (11)
Tiihonen et al. (2008)	Introduction, results, and discussion text, tables	FDR (*p* <0.05) for all ROIs; whole brain analyses of gray and white matter plus focal ROIs	66	56.06% (37)	43.94% (29)
Craig et al. (2009)	Results text, tables	Bonferroni correction for multiple comparisons (stream lines and FA)	4	25% (1)	75% (3)
Yang et al. (2009a)	Text	Permutation corrected	4	50% (2)	50% (2)
Yang et al. (2009b)	Text	Permutation corrected	4	100% (4)	0% (0)
Yang et al. (2010)	Introduction and results text, tables	Multivariate correction for confounds	48	16.67% (8)	83.33% (40)
Boccardi et al. (2010)	Introduction and results text, tables	Permutation corrected	12	25% (3)	75% (9)
Glenn et al. (2010a)	Introduction and results text	Multivariate statistical control for confounds	7	42.86% (3)	57.14% (4)
Glenn et al. (2010b)	Introduction and results text	Multivariate statistical control for confounds	3	0% (0)	100% (3)
Raine et al. (2010)	Results text	Multivariate correction for confounds	1	100% (1)	0% (0)
Boccardi et al. (2011)	Introduction, results, and discussion text, tables, Supplementary materials	Results taken as reported by authors in supplementary tables (40 ROIs for all pair-wise comparisons of three groups)	312	41.67% (130)	58.33% (182)
Motzkin et al. (2011)	Results text	Group comparison for single structure	2	50% (1)	50% (1)
Schiffer et al. (2011)	Introduction and results text, tables	FDR (*p* < 0.05) for whole-brain analysis	8	62.5% (5)	37.5% (3)
Yang et al. (2011)	Introduction and results text	Permutation corrected	4	75% (3)	25% (1)
Cope et al. (2012)	Introduction and results text, tables	Small volume correction, FWE (*p* < 0.05)	26	3.85% (1)	96.15% (25)
Ermer et al. (2012)	Introduction and results text, tables	FDR correction for peak height analyses and cluster extent analyses	60	20% (12)	80% (48)
Gregory et al. (2012)	Introduction and results text, tables	FDR correction for cluster extent analyses, controlling for confounds	20	20% (4)	80% (16)
Howner et al. (2012)	Introduction and results text, tables	FDR correction for thickness maps	11	27.27% (3)	72.73% (8)
Ly et al. (2012)	Introduction and results text, tables	Cluster extent thresholding (uncorrected *p* < 0.005)	13	100% (13)	0% (0)
Boccardi et al. (2013)	Introduction and results text, tables	Permutation corrected	18	16.67% (3)	83.33% (15)
Pujara et al. (2013)	Introduction and results text	Tissue segmentation	8	0% (0)	100% (8)
Sethi et al. (2015)	Introduction and results text, tables	Statistical control for confounds ANOVA	4	25% (1)	75% (3)
Wolf et al. (2015)	Introduction and results text	Multivariate control for confounds	2	50% (1)	50% (1)
Contreras-Rodríguez et al. (2015)	Introduction and results text, Supplementary tables	FWE correction (*p* < 0.05)	19	100% (19)	0% (0)
Korponay et al. (2017a)	Introduction and results text, tables	Peak height FWE correction (*p* < 0.05)	7	14.29% (1)	85.71% (6)
Korponay et al. (2017b)	Introduction and results text, supplementary text and tables	Small volume correction	8	62.5% (5)	37.5% (3)
Lam et al. (2017)	Introduction and results text, tables	Multivariate correction for multiple comparisons	12	33.33% (4)	66.67% (8)
Crooks et al. (2018)	Introduction and results text, tables	Spearman's rho; partial correlations (to control for confounds)	1	100% (1)	0% (0)
Miskovich et al. (2018)	Introduction and results text	Cluster correction for multiple comparisons (*p* < 0.05)	4	50% (2)	50% (2)
Crooks et al. (2019)	Introduction and results text, tables	Spearman's rho; partial correlations and regression (to control for confounds)	1	0% (0)	100% (1)
Total			791	35.90% (284)	64.1% (507)

### Proportion of Null-Findings in Review Literature

We included 45 relevant publications, of which 43 were review studies and two were meta-analyses. Overall, of the 1,001 effects reported in the review literature, 8.99% (*N* = 90) were null-findings. The remaining 91.01% (*N* = 911) were positive findings (see Table 2). The difference between the proportion of null-findings in original studies and review literature was statistically significant (χ^2^ = 1321.07, *p* < 0.00001).

**Table 2:** Summary of review study effects.

**Review studies**	**Type of review*^a^***	**sMRI studies reviewed**	**Number of effects**		
			**Reject % (N)**	**Null % (N)**	**Applied Y/N**
Bassarath (2001)	N	Laakso et al. (2001)	100% (2/2)	0% (0/2)	N
Blair (2003)	E	Raine et al. (2000)	50% (1/2)	50% (1/2)	N
Pridmore et al. (2005)	N	Raine et al. (2000), Laakso et al. (2002), Laakso et al. (2001), Raine et al. (2003)	57% (4/7)	43% (3/7)	N
Anckarsäter (2006)	N	Laakso et al. (2001, 2002), Raine et al. (2003)	80% (4/5)	20% (1/5)	N
Kiehl (2006)	T	Laakso et al. (2001), Raine et al. (2004)	100% (2/2)	0% (0/2)	N
Raine and Yang (2006a)	N	Raine et al. (2000), Yang et al. (2005), Laakso et al. (2002), Raine et al. (2004), Laakso et al. (2001)	100% (7/7)	0% (0/7)	Y
Raine and Yang (2006b)	N	Raine et al. (2000), Laakso et al. (2001), Raine et al. (2004), Raine et al. (2003), Raine et al. (2004), Yang et al. (2005)	73% (8/11)	27% (3/11)	Y
Herba et al. (2007)	T	Laakso et al. (2002), Raine et al. (2004), Raine et al. (2003)	50% (5/10)	50% (5/10)	N
Glenn and Raine (2008)	N	Raine et al. (2004), Laakso et al. (2001), Raine et al. (2000), Yang et al. (2005)	100% (4/4)	0% (0/4)	N
Weber et al. (2008)	N	Raine et al. (2000), Yang et al. (2005), Laakso et al. (2002), Müller et al. (2008), Laakso et al. (2001), Raine et al. (2004), Raine et al. (2003)	64% (9/14)	36% (5/14)	Y
Yang et al. (2008)	N	Laakso et al. (2001), Raine et al. (2003), Raine et al. (2004), Yang et al. (2005)	100% (6/6)	0% (0/6)	Y
Gao et al. (2009)	N	Raine et al. (2000), Yang et al. (2005), Müller et al. (2008), de Oliveira-Souza et al. (2008), Laakso et al. (2001), Raine et al. (2004), Raine et al. (2003)	82% (9/11)	18% (2/11)	N
Plodowski et al. (2009)	N	Raine et al. (2000), Raine et al. (2003), Yang et al. (2005), Raine et al. (2004), Laakso et al. (2002), Laakso et al. (2001), de Oliveira-Souza et al. (2008)	54% (21/39)	46% (18/39)	N
Wahlund and Kristiansson (2009)	T	Laakso et al. (2001), Raine et al. (2000), Laakso et al. (2002), Yang et al. (2005), Raine et al. (2003), Raine et al. (2004)	89% (8/9)	11% (1/9)	N
Yang and Raine (2009)	M	Laakso et al. (2002), Raine et al. (2000)	100% (2/2)	0% (0/2)	N
Blair (2010)	T	Yang et al. (2009b), Raine et al. (2004), Laakso et al. (2001), Raine et al. (2003), Glenn et al. (2010a), Tiihonen et al. (2008), de Oliveira-Souza et al. (2008), Müller et al. (2008)	100% (14/14)	0% (0/14)	N
Muller (2010)	N	Tiihonen et al. (2008), Yang et al. (2005), Laakso et al. (2002), Raine et al. (2000), Müller et al. (2008), Raine et al. (2004), Laakso et al. (2001), Raine et al. (2003)	80% (16/20)	20% (4/20)	N
Koenigs et al. (2011)	N	Yang et al. (2005), Yang et al. (2010), Ermer et al. (2012), Yang et al. (2009b), Boccardi et al. (2011), Ly et al. (2012), Müller et al. (2008), Craig et al. (2009), Motzkin et al. (2011)	89% (17/19)	11% (2/19)	N
Anderson and Kiehl (2012)	N	Ermer et al. (2012), Yang et al. (2010), Boccardi et al. (2011), Tiihonen et al. (2008), de Oliveira-Souza et al. (2008), Boccardi et al. (2010), Müller et al. (2008)	100% (27/27)	0% (0/27)	Y
Koenigs (2012)	T	Yang et al. (2009a), Müller et al. (2008), Yang et al. (2010), Raine et al. (2003), Laakso et al. (2001), Boccardi et al. (2010), Craig et al. (2009)	90% (9/10)	10% (1/10)	N
Blair (2013)	T	Ermer et al. (2012), Yang et al. (2010), Yang et al. (2009a), Gregory et al. (2012), Ly et al. (2012), de Oliveira-Souza et al. (2008), Craig et al. (2009), Motzkin et al. (2011)	100% (14/14)	0% (0/14)	N
Loomans et al. (2013)	N	Raine et al. (2000), Yang et al. (2005), Tiihonen et al. (2008), Müller et al. (2008), Raine et al. (2003), Laakso et al. (2001), Raine et al. (2004)	92% (22/24)	8% (2/24)	N
Anderson and Kiehl (2014a)	N	Ermer et al. (2012), Yang et al. (2010), Boccardi et al. (2011), Tiihonen et al. (2008), de Oliveira-Souza et al. (2008), Yang et al. (2011), Müller et al. (2008)	100% (16/16)	0% (0/16)	Y
Anderson and Kiehl (2014b)	N	Boccardi et al. (2011), Yang et al. (2010), Müller et al. (2008), Yang et al. (2011), de Oliveira-Souza et al. (2008), Ermer et al. (2012)	100% (11/11)	0% (0/11)	N
Aoki et al. (2014)	M	de Oliveira-Souza et al. (2008), Gregory et al. (2012), Tiihonen et al. (2008)	100% (3/3)	0% (0/3)	N
Debowska et al. (2014)	N	Yang et al. (2005), Yang et al. (2010), Gregory et al. (2012), Boccardi et al. (2011), Laakso et al. (2001), Yang et al. (2009b), Müller et al. (2008), Craig et al. (2009), Motzkin et al. (2011)	88% (22/25)	12% (3/25)	N
Glenn and Raine (2014)	N	Yang et al. (2005), Yang et al. (2009b), Müller et al. (2008), de Oliveira-Souza et al. (2008), Boccardi et al. (2010), Raine et al. (2004), Laakso et al. (2001), Glenn et al. (2010a), Glenn et al. (2010b), Raine et al. (2003), Craig et al. (2009)	96% (24/25)	4% (1/25)	Y
Patrick (2014)	N	Müller et al. (2008), Yang et al. (2005), Yang et al. (2009b), Raine et al. (2004), Boccardi et al. (2010), Raine et al. (2003), Glenn et al. (2010a), Craig et al. (2009), Glenn et al. (2010b), Gregory et al. (2012)	88% (15/17)	12% (2/17)	N
Pujara and Koenigs (2014)	N	Boccardi et al. (2011), Ermer et al. (2012), Yang et al. (2009b), Yang et al. (2010), de Oliveira-Souza et al. (2008), Gregory et al. (2012), Ly et al. (2012), Müller et al. (2008), Yang et al. (2005), Yang et al. (2011), Glenn et al. (2010a,b), Pujara et al. (2013), Raine et al. (2003), Laakso et al. (2001), Boccardi et al. (2010), Raine et al. (2010), Craig et al. (2009), Motzkin et al. (2011)	98% (41/42)	2% (1/42)	N
Stratton et al. (2015)	N	Contreras-Rodríguez et al. (2015), Ermer et al. (2012), Yang et al. (2009a), Yang et al. (2009b), Boccardi et al. (2011), de Oliveira-Souza et al. (2008), Tiihonen et al. (2008), Cope et al. (2012), Ly et al. (2012), Glenn et al. (2010a), Pujara et al. (2013), Craig et al. (2009), Motzkin et al. (2011)	100% (42/42)	0% (0/42)	Y
Umbach et al. (2015)	N	Yang et al. (2009b), Boccardi et al. (2011), Ermer et al. (2012), Yang et al. (2010), Gregory et al. (2012), de Oliveira-Souza et al. (2008), Howner et al. (2012), Craig et al. (2009), Schiltz et al. (2007)	86% (18/21)	14% (3/21)	Y
Lushing et al. (2016)	N	Boccardi et al. (2010), Boccardi et al. (2011), Cope et al. (2012), Ermer et al. (2012), Tiihonen et al. (2008), Boccardi et al. (2013), Contreras-Rodríguez et al. (2015), Gregory et al. (2012), Ly et al. (2012), Laakso et al. (2001)	96% (52/54)	4% (2/54)	Y
Santana (2016)	S	Tiihonen et al. (2008), Howner et al. (2012), Boccardi et al. (2011), Raine et al. (2000), Yang et al. (2005), Yang et al. (2009a), Yang et al. (2009b), Laakso et al. (2002), de Oliveira-Souza et al. (2008), Ermer et al. (2012), Cope et al. (2012), Müller et al. (2008), Ly et al. (2012), Gregory et al. (2012), Glenn et al. (2010a,b), Schiffer et al. (2011), Laakso et al. (2001), Raine et al. (2004), Boccardi et al. (2010), Raine et al. (2003), Craig et al. (2009), Motzkin et al. (2011)	91% (86/95)	9% (9/95)	N
Smith et al. (2016)	T	Boccardi et al. (2011), Boccardi et al. (2013), Tiihonen et al. (2008)	100% (14/14)	0% (0/14)	Y
Ortega-Escobar et al. (2017)	T	Gregory et al. (2012), Ermer et al. (2012), Boccardi et al. (2011), Boccardi et al. (2013), Yang et al. (2010), Motzkin et al. (2011), Craig et al. (2009)	100% (20/20)	0% (0/20)	Y
Gao (2018)	N	Raine et al. (2000), Yang et al. (2005), Yang et al. (2009a), Yang et al. (2009b), Müller et al. (2008), de Oliveira-Souza et al. (2008), Gregory et al. (2012), Laakso et al. (2001), Yang et al. (2010), Ermer et al. (2012), Howner et al. (2012), Raine et al. (2003), Glenn et al. (2010a), Raine et al. (2004), Motzkin et al. (2011), Sethi et al. (2015), Craig et al. (2009), Wolf et al. (2015)	90% (37/41)	10% (4/41)	N
Ling and Raine (2018)	T	Yang et al. (2009a), Yang et al. (2009b), Ermer et al. (2012), de Oliveira-Souza et al. (2008), Ly et al. (2012), Yang et al. (2011), Ermer et al. (2012), Yang et al. (2005), Craig et al. (2009), Motzkin et al. (2011), Wolf et al. (2015), Glenn et al. (2010a), Cope et al. (2012), Korponay et al. (2017b)	91% (21/23)	9% (2/23)	Y
Ling et al. (2018)	T	de Oliveira-Souza et al. (2008), Müller et al. (2008), Ly et al. (2012), Ermer et al. (2012), Craig et al. (2009), Motzkin et al. (2011), Yang et al. (2009a), Yang et al. (2009b), Yang et al. (2011), Boccardi et al. (2013), Glenn et al. (2010a), Schiffer et al. (2011), Cope et al. (2012), Yang et al. (2010), Yang et al. (2005), Gregory et al. (2012), Korponay et al. (2017b), Raine et al. (2004)	92% (48/52)	8% (4/52)	N
Murray et al. (2018)	N	Ermer et al. (2012), Glenn et al. (2010a), Korponay et al. (2017b), Motzkin et al. (2011)	100% (7/7)	0% (0/7)	N
Pujol et al. (2018)	N	Yang et al. (2005), Tiihonen et al. (2008), Contreras-Rodríguez et al. (2015), Raine et al. (2010), Laakso et al. (2002), de Oliveira-Souza et al. (2008), Müller et al. (2008), Yang et al. (2009a), Yang et al. (2009b), Yang et al. (2010), Gregory et al. (2012), Ly et al. (2012), Ermer et al. (2012), Boccardi et al. (2011), Laakso et al. (2001), Raine et al. (2004), Glenn et al. (2010a,b), Boccardi et al. (2013), Raine et al. (2003), Craig et al. (2009), Motzkin et al. (2011), Sethi et al. (2015), Pujara et al. (2013), Wolf et al. (2015)	92% (82/89)	8% (7/89)	N
Yang and Raine (2018)	N	Yang et al. (2010), Yang et al. (2005), Yang et al. (2011), Cope et al. (2012), Ermer et al. (2012), Raine et al. (2000), Gregory et al. (2012), Yang et al. (2009a), Yang et al. (2009b), Contreras-Rodríguez et al. (2015), Ly et al. (2012), Boccardi et al. (2011), Boccardi et al. (2010), Raine et al. (2004), Schiffer et al. (2011), Glenn et al. (2010a), Boccardi et al. (2013), Raine et al. (2003), Craig et al. (2009)	96% (48/50)	4% (2/50)	Y
Moreira et al. (2019)	S	Gregory et al. (2012), Yang et al. (2009b), Laakso et al. (2001)	100% (9/9)	0% (0/9)	N
Raine (2019)	T	Glenn et al. (2010a)	100% (4/4)	0% (0/4)	Y
Blair and Zhang (2020)	T	Crooks et al. (2018), Craig et al. (2009), Wolf et al. (2015)	100% (3/3)	0% (0/3)	N
Johanson et al. (2020)	S	Boccardi et al. (2010), Boccardi et al. (2011), Boccardi et al. (2013), Contreras-Rodríguez et al. (2015), Cope et al. (2012), de Oliveira-Souza et al. (2008), Ermer et al. (2012), Glenn et al. (2010a), Glenn et al. (2010b), Gregory et al. (2012), Howner et al. (2012), Korponay et al. (2017a), Korponay et al. (2017b), Laakso et al. (2002), Ly et al. (2012), Müller et al. (2008), Raine et al. (2003), Raine et al. (2004), Tiihonen et al. (2008), Yang et al. (2009b), Yang et al. (2010)	96% (78/81)	4% (3/81)	N
**Total**			91.01% (911/1001)	8.99% (90/1001)	Y 33% (15/45) N 67% (30/45)

a *C, comprehensive; E, editorial; M, meta-analysis; N, narrative (including reviews described as “critical”); S, systematic; T, targeted/focused*.

In order to exclude the possibility that something other than coding bias can explain the discrepancy, we considered the possibility that reviews focused on theoretically important regions could have yielded more positive findings than theoretically peripheral areas. We ran two additional analyses: First, to account for the possibility that a disproportionate number of null-findings came from exploratory, whole-brain analyses of theoretically unrelated regions, we repeated the analysis of the sMRI research excluding studies whose authors identified them as exploratory (these studies were Müller et al., 2008; Tiihonen et al., 2008; Howner et al., 2012; Contreras-Rodríguez et al., 2015). This analysis yielded 67.25% (*N* = 460) null-findings. In other words, the proportion of null-findings did not appear to be driven by exploratory studies reporting on areas not theorized to be relevant to psychopathy.

Second, we reviewed citation patterns at the effect level. We focused on the amygdala, because it is (a) central to prevailing neurobiological theories of psychopathy and thus widely cited in the review literature (Kiehl, 2006; Blair, 2008), and (b) narrowly and consistently defined across original and review literature, permitting a direct focal comparison between the two types of literatures. The original sMRI studies reported 13 results for the amygdala: six null-findings (Schiltz et al., 2007; de Oliveira-Souza et al., 2008; Tiihonen et al., 2008; Cope et al., 2012; Ermer et al., 2012; Gregory et al., 2012), four volumetric reductions (Yang et al., 2009b, 2010; Ermer et al., 2012; Contreras-Rodríguez et al., 2015, one enlargement (Boccardi et al., 2011), one non-linear PCL-R and volume correlation (Schiffer et al., 2011), and one difference in surface shape (Yang et al., 2010).^3^ The percentage of null-findings thus accounted for 46.15% of the findings. In contrast, review studies reported 116 findings for the amygdala, of which three (2.59%) were null-findings. Therefore, low proportions of null-findings cannot be attributed to reviewers documenting positive findings in theoretically salient regions and ignoring peripheral noise in the sMRI literature.

**Figure 2:**
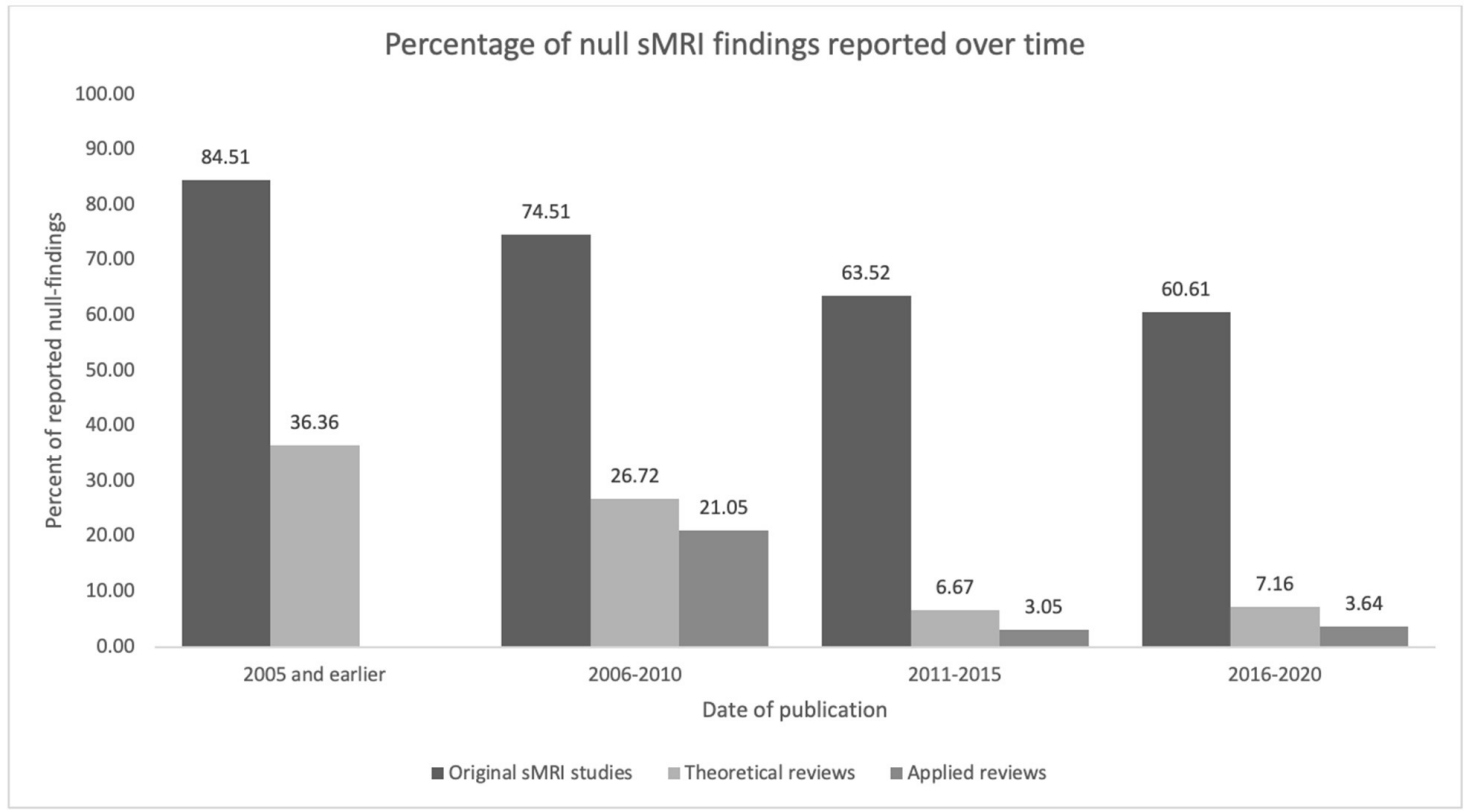
Null sMRI findings reported over time.

To account for the possibility that some reviewers might report fewer null-findings simply because the prevalence of null-findings has changed over time (i.e., perhaps earlier original research reported fewer null-findings than later research), we examined the proportion of null-findings in the original sMRI studies at 5 year intervals. As is apparent in Figure 2 and Table 3, the proportion of nulls has decreased with time in both original studies and review literature [the trend appears similar to that in neuroscientific literature in general; see Fanelli (2012)]. Therefore, chronological changes or study availability do not appear to explain our results.

**Table 3:** Null sMRI findings reported over time.

**Date of publication**	**Type of study**	**Total number of sMRI effects**	**N nulls reported (% of Total)**	**N positive findings reported (% of total)**
2005 and earlier	Original sMRI	71	60 (84.51%)	11 (15.49%)
	Theoretical reviews	11	4 (36.36%)	7 (63.64%)
	Applied reviews	0	0	0
2006–2010	Original sMRI	102	76 (74.51%)	26 (25.49%)
	Theoretical reviews	126	31 (26.72%)	85 (73.28%)
	Applied reviews	38	8 (21.05%)	30 (78.95%)
2011–2015	Original sMRI	477	303 (63.52%)	174 (36.48%)
	Theoretical reviews	165	11 (6.67%)	154 (93.33%)
	Applied reviews	131	4 (3.05%)	127 (96.95%)
2016–2020	Original sMRI	33	20 (60.61%)	13 (39.39%)
	Theoretical reviews	377	27 (7.16%)	350 (92.84%)
	Applied reviews	165	6 (3.64%)	159 (96.36%)

Finally, to ensure that we did not include original studies that reviewers had designated as irrelevant we compared our list of original studies to studies cited in the review literature. All the original studies in our list were cited at least once in the review literature (see Table 2).

### Proportion of Null-Findings by Review Type

We classified reviews according to their study design. We identified two meta-analyses, three systematic reviews, 27 narrative reviews, 12 targeted/focused reviews^4^, and one editorial. The meta-analyses included only five effects and the editorial included two. The remaining three categories included much larger number of effects: systematic reviews (*n* =185), narrative reviews (*n* = 636), and targeted reviews (*n* = 175). We examined whether reviews using a systematic search strategy reported more null-findings than reviews using other designs. Narrative reviews were more likely to cite null-findings than systematic or targeted reviews [χ^2^ (2, *N* = 996) = 14.87, *p* < 0.001]. However, the difference was entirely driven by a single narrative review (Plodowski et al., 2009) that cited an accurate proportion of null-findings. After removing this outlier, there was no difference in the proportion of nulls by review type [χ^2^ (2, *N* = 957) = 2.15, *p* = *ns*]. That is, reviews using systematic database searches were no less subject to coding bias than other types of reviews.

We also classified reviews into those that made forensic recommendations (applied reviews) and those that did not (theoretical reviews). Theoretical reviews (*N* = 30) reported 669 effects, of which 10.91% (*N* = 73) were null-findings and 89.09% (*N* = 596) were positive findings. Applied reviews (*N* = 15) reported 334 effects, of which 5.39% (*N* = 18) were null-findings and 94.61% (*N* = 316) were positive findings. The difference between applied and theoretical reviews was statistically significant (χ^2^ = 10.47, *p* < 0.01). One outlier (Plodowski et al., 2009), however, reported 24.7% (*N* = 18) of the 73 null-findings in theoretical reviews. After removing this outlier, the discrepancy was no longer significant (χ^2^ = 2.84, *p* = *ns*).

Finally, we examined the proportion of review studies that found support for neurobiological bases of psychopathy. Twenty-one of the 30 theoretical reviews (70%) found general support for the neurobiological bases of psychopathy while four studies found the data to be inconclusive (Herba et al., 2007; Muller, 2010; Koenigs et al., 2011; Pujara and Koenigs, 2014). One meta-analysis (Yang and Raine, 2009) examined whether PCL-R scores moderated the relationship between antisocial behavior and prefrontal volumes, and found that they did not. The studies that found the data to be inconclusive did so based on (a) the widespread nature of the findings and/or (b) the fact that the positive findings included both increased and decreased volume. Three studies reached only tentative conclusions (Plodowski et al., 2009; Wahlund and Kristiansson, 2009; Santana, 2016), and one meta-analysis (Aoki et al., 2014) did not report findings on psychopathy separately from general antisocial traits and behaviors. In contrast, all 15 applied reviews interpreted the data to indicate neurobiological bases of psychopathy.

## Discussion

Neurobiological reviews of PCL-R and PCL:SV psychopathy significantly under-report null-findings in sMRI research, indicating widespread coding bias. The majority (64.18%) of original sMRI findings were nulls, whereas nulls made up a small minority (8.99%) of effects in review literature. Reviewers, in other words, preferentially reported data supporting neurobiological models of psychopathy. We found no evidence that the reporting imbalance was due to factors other than bias: systematic, narrative, and targeted reviews all reported disproportionately few nulls (though meta-analyses reported too few effects to evaluate), the pattern was stable across time, and not driven by exploratory research or outliers. Notably, reviews calling for forensic application of the data, such as treatment, criminal responsibility, punishment, and crime prediction, were no more accurate than purely theoretical reviews. Applied reviews were, however, more likely than theoretical reviews to conclude that the data supported neurobiological bases of psychopathy. These findings are surprising, as applied reviews in other fields—such as those examining drug safety and efficacy—typically face the highest burden of proof and are thus most likely to emphasize limitations in the data [see e.g., Köhler et al. (2015)].

Our study is the first to systematically examine coding bias in cognitive neuroscience. Although our findings are limited to structural imaging in psychopathy, they suggest that coding bias should be considered alongside more widely recognized Questionable Research Practices (QRPs) such as p-hacking, reporting bias, publication bias, citation bias, and the file drawer problem. QRPs in original research filter out null-findings at early stages of the research and publication process, while coding and citation bias further distort the state of scientific knowledge by eliminating null findings from reviews. In addition to coding bias, we found evidence of reporting bias during our review of sMRI studies. Null-findings in the original literature were rarely reported in the study abstracts and were frequently not reported fully in results sections. Nulls often appeared only in data or supplemental tables, and in some cases they had to be inferred by examining ROIs mentioned in the introduction but not in the results section. This illustrates how QRPs are not mutually exclusive, and the presence of one QRP may also signal the presence of another [see e.g., Agnoli et al. (2017)].

The coding bias we observed may have a number of explanations. First, reviewers may have been subject to confirmation bias. Confirmation bias refers to the tendency to weigh evidence that confirms a belief more heavily than evidence that does not (Nickerson, 1998). Reviewers in our study may have assumed neurobiological abnormalities in psychopaths—perhaps from previous reviews—and looked more carefully for data to confirm that assumption. Confirmation bias has been cited as a possible explanation for under-reporting of null-findings in original research (Forstmeier et al., 2017). Our findings suggest that it may play a role in review literature, where null-findings would be especially difficult to square with theories presuming group differences [see e.g., Sterling et al. (1995) and Ferguson and Heene (2012)], and reporting bias would make it very hard to locate disconfirming (null) findings. Second, reviewers may have been following convention. The earliest review studies did not generally include null-findings, and later reviews may have interpreted this as a precedent to follow. Third, explicit and tacit publication preferences may increase coding bias. Research tracking original studies from grant proposal to publication show that most null-findings are not even written up for publication, and that journals—particularly top-tier journals—show a marked preference for strong positive findings (Franco et al., 2014; Ioannidis et al., 2014). Similarly, review authors may have declined to submit reviews with inconclusive findings. Given the extent of publication bias, it is also possible that journal editors may have been more likely to reject inconclusive reviews in favor of those summarizing consistent, positive findings.

Coding bias observed in our study has a number of potential effects. Aside from distorting the true state of knowledge about structural brain abnormalities in psychopaths, it may also have led at least some researchers and courts to believe that the abnormalities are consistent enough for forensic application. This may have encouraged practitioners to de-emphasize or overlook more reliable, behavioral indicators of criminal responsibility, future dangerousness and treatment amenability in favor of less reliable predictors, such as brain structure. Neuroprediction of crime has a number of empirical shortcomings, such as unknown measurement error and inadequate outcome variables (Poldrack et al., 2018). Using MRI data to predict crime can thus introduce substantial error into an already imperfect process (e.g., Douglas et al., 2017). Neurobiologically-informed assessments and treatments are even less likely to be effective if the population's neurobiology is fundamentally misunderstood. Given the extent of coding bias in the psychopathy literature, such interventions may in fact be harmful.

More broadly, coding bias may have contributed to reverse inference [see Scarpazza et al. (2018)] whereby reports of brain abnormalities are taken as proof that psychopathy is a legitimate diagnostic category [for an argument such as this, see e.g., Kiehl and Hoffman (2011)].^5^ Similarly, some researchers have suggested that psychopathy diagnoses could be enhanced by neuroimaging evidence (e.g., Hulbert and Adeli, 2015). Arguments of this sort can detract from problems in other aspects of the PCL-R, particularly in its psychometric properties. Recently, these critiques have intensified, with authors raising concerns about the reliability of the PCL-R, its utility in forensic contexts (DeMatteo et al., 2020), its factor structure, and its predictive validity (Boduszek and Debowska, 2016). Using neurobiology to validate psychopathy as a diagnostic category is doubly problematic: not only are presumed brain abnormalities in psychopathy broad and non-specific [for problems in reverse inference, see Poldrack (2011) and Scarpazza et al. (2018)], but as we have shown here, their consistency appears to be largely misunderstood as well.

In light of our findings, we recommend the following: First, published review literature on sMRI studies of PCL-R and PCL:SV psychopathy should be approached with caution, especially when the literature is used to influence forensic decisions. Second, we recommend that guidelines for conducting review literature be revised to include explicit guidance for avoiding coding bias. Although the problem of un- and under-reported null-findings is recognized [e.g., Pocock et al., 1987; Hutton and Williamson, 2000; guidelines for accurate reporting in review literature also exist; see Petticrew and Roberts (2008), American Psychological Association (2008), and Moher et al. (2015)], the role of coding bias, by and large, is not. Third, we recommend that review literature pay careful attention to the *a priori* likelihood of null-findings in their data. In our example, both the PCL-R (DeMatteo et al., 2020) and neuroimaging methods (Nugent et al., 2013) have relatively low reliability. The likelihood that sMRI research on psychopathy should yield more than 91% positive findings is therefore not realistic [for more extended discussions relating to fMRI, see Vul et al. (2009) and Vul and Pashler (2017)]. Fourth, we recommend that the production of new data should be complemented by closer examination of data already published. Among the 45 reviews we evaluated, we found a single study (Plodowski et al., 2009) that comprehensively reported all nulls in the original literature. Unfortunately, it was also among the least cited reviews, suggesting that accuracy and scientific impact do not necessarily go together. Finally, we recommend that reviewers pay close attention to potential biases—such as publication and reporting bias, p-hacking, and the file drawer problem—in the original literature, and take measures to compensate for them. Currently, it appears that reviews largely magnify them instead.

## Limitations

Our study has a number of important limitations. First, in order to focus on forensically relevant studies, we limited our analysis to PCL-R and PCL:SV psychopathy. We also excluded studies that reported on PCL-R Factor scores only (e.g., Bertsch et al., 2013), that did not use case-control or correlational method (Sato et al., 2011; Kolla et al., 2014), and that included youth samples. It is possible that the excluded studies were reported more accurately in review literature than those we included. Second, we excluded original and review studies not published in English. This may have introduced a selection bias of our own, as it is possible that non-English publications use different standards of reporting and reviewing than those published in English. Third, our findings may have underestimated the extent of the bias. For example, one whole-brain analysis reviewed here (Contreras-Rodríguez et al., 2015) only reported positive findings, which means that the remaining brain regions were unreported nulls. Had these unreported null-findings been included in our analysis, the true percentage of nulls in the original studies would have been greater than 64.18%. Further, we did not account for possible publication bias. Since null-findings are presumed to be less likely than null-rejections to be published, the percentage of true nulls in the field is essentially unknown, though it may be significantly higher than we estimated (review literature examined here did not report any unpublished null-findings). Finally, we excluded fMRI and other imaging methods entirely. Future research could evaluate whether coding bias is present in reviews of this literature as well.

## Data Availability Statement

The original contributions presented in the study are included in the article/Supplementary Material, further inquiries can be directed to the corresponding author/s.

## Author Contributions

Topic conceptualization was completed by JJ, SG, and RL. The PRISMA review was conducted by RL and JJ. Effect coding was conducted by JJ, SG, and BA. Coding disagreements were resolved by RL. Data analysis completed by JJ and SG. Manuscript preparation was completed by JJ, with edits from SG and RL. All authors contributed to the article and approved the submitted version.

## Conflict of Interest

The authors declare that the research was conducted in the absence of any commercial or financial relationships that could be construed as a potential conflict of interest.

## Footnotes

^1^At this step the excluded articles were Baskin-Sommers et al. (2016), Harris et al. (2001), Hoppenbrouwers et al. (2015), Kolla et al. (2014), Kolla et al. (2017), Pera-Guardiola et al. (2016), Yang et al. (2012), Beckwith et al. (2018), Bertsch et al. (2013), Leutgeb et al. (2015), Vermeij et al. (2018), Calzada-Reyes et al. (2015), Korponay (2019), Vieira et al. (2015), and Yang (2009).

^2^At this step the excluded articles were Nickerson (2014), Blair (2019), Brower and Price (2001), Dolan (2002), Lamsma et al. (2017), and Raine (2019).

^3^Ermer et al. (2012) found both a reduction (using cluster size) and a null (using peak height). We are thus reporting it twice in this analysis.

^4^Targeted/focused reviews included data only on specific brain region(s) or outcome(s) (e.g., antisocial behavior).

^5^Though, Kiehl and Hoffman (2011) refer mostly to functional MRI data, their argument is a classic example of reverse inference. They write: “(T)hese neurological results should go a long way toward ending the debate about whether psychopathy is just too difficult to diagnose to justify inclusion in the DSM. Any lingering doubts about the clinical reliability of the Hare instruments disappear now that those instruments have been shown to be robustly predictive of a demonstrable neurological condition.” (p. 390).
