# Supplementary material for: Is the Psychopathic Brain an Artifact of Coding Bias? A Systematic Review
Source: Front Psychol. 2021 Apr 12;12:654336. doi: 10.3389/fpsyg.2021.654336 (PMC8071952; doi:10.3389/fpsyg.2021.654336)
Supplement: Supplementary file 1 [file Data_Sheet_1.docx]

Appendix

1. Find the sMRI effects in the review literature. Refer to the section of the article entitled “structural findings” or that clearly report structural MRI effects using terms such as “size” and/or “volume” of structures, “grey matter”, and related terms.
2. Find the source of reported sMRI effects. Note the authorship of reported sMRI effects (for example, “from Ermer et al., 2012”).
3. Describe the effect. Code each sMRI effect as:
   1. Positive (null rejection) vs null-finding

| ***Positive findings*** | ***Null-finding*** |
| --- | --- |
| Larger, bigger, increased, smaller, decreased, atrophy, abnormality, significant difference, difference, ↑↓, > <, + or - | Same, no difference, comparable, null, same as controls |

- 1. Bilateral or unilateral (Right or left sided).

| ***Bilateral*** | ***Unilateral*** |
| --- | --- |
| Both, pluralized structure name (e.g. “hippocampi”, “amygdalae”, “cortices”, and “nuclei” are plural).  CODE AS **2 EFFECTS**– EITHER POSITIVE OR NULL AS THE CASE MAY BE | Left or right, one-sided, (e.g., “hippocampus”, “amygdala”, “cortex”, and nucleus” are unilateral).  CODE AS **1 EFFECT** – EITHER POSITIVE OR NULL |

Vague, diffuse, or broad spatial descriptions (e.g., prefrontal) are coded as a single effect, even though that region includes multiple foci. In the case of pluralized effects from a single study, such as “prefrontal regions”, two effects are coded. In the case of pluralized findings from multiple sources, such as “prefrontal regions” from 3 original studies, a single effect is coded from each original study.

- 1. Some “sMRI studies” sections contain multiple effects, which are usually reported in one of two ways.
     1. By original study. One section (table or text) summarizes all effects from each sMRI study being reviewed. For example, “Raine et al., 2003 found smaller hippocampi, left amygdala, and bilateral cingulate structures” – this would be coded as 5 positive effects (i.e., 2 hippocampi, 1 amygdala, 2 cingulate). Another example, “Glenn et al., 2010 found reduced size and grey matter density in bilateral cingulate cortices” would be coded as 4 positive findings (2 for size and 2 for density).
     2. By region. Effects from multiple sMRI studies are integrated into discussions of specific brain regions (e.g. the amygdalae). Count the number of effects cited from each original sMRI study throughout the review. For example, Ermer et al. (2012) effects may appear in several sections (e.g., smaller amygdala, parahippocampus, and orbitofrontal cortex) – these citations need to be added into one number (in this example, record 3 positive findings for Ermer et al., 2012).
  2. Focus on data summary tables. Some Reviews describe sMRI studies in the text, some in tables, and some in both. Tables are the primary source. However, check the text as well, as sometimes more information is added there, but make sure not to double-count the findings.
  3. Code only for PCL-R Total score. If one of the Reviews describes “sMRI study” findings by Factors (1 or 2) of facets (1-4) of the PCL-R, disregard these. Only code findings for PCL-R total score (described as “psychopathy”, “psychopaths” or “PLC-R”). For example, disregard tables of structural correlates of “affective-interpersonal dimensions” of psychopathy (e.g., Johanson et al., 2020).
  4. Code all relevant group comparisons. For example, some original sMRI studies distinguish between “successful” and “unsuccessful” psychopaths. Code findings for these groups separately. For example, if the review says “unsuccessful psychopaths showed smaller amygdala volume”, code this as one positive finding. If it says “unsuccessful, but not successful psychopaths, showed smaller amygdala volume”, code this as one positive finding and one null.
